# Supplementary material for: The cryo-EM structure of hibernating 100S ribosome dimer from pathogenic Staphylococcus aureus
Source: Nat Commun. 2017 Sep 28;8:723. doi: 10.1038/s41467-017-00753-8 (PMC5620080; doi:10.1038/s41467-017-00753-8)
Supplement: Supplementary file 1 — Supplementary Information [file 41467_2017_753_MOESM1_ESM.pdf]

Supplementary Information

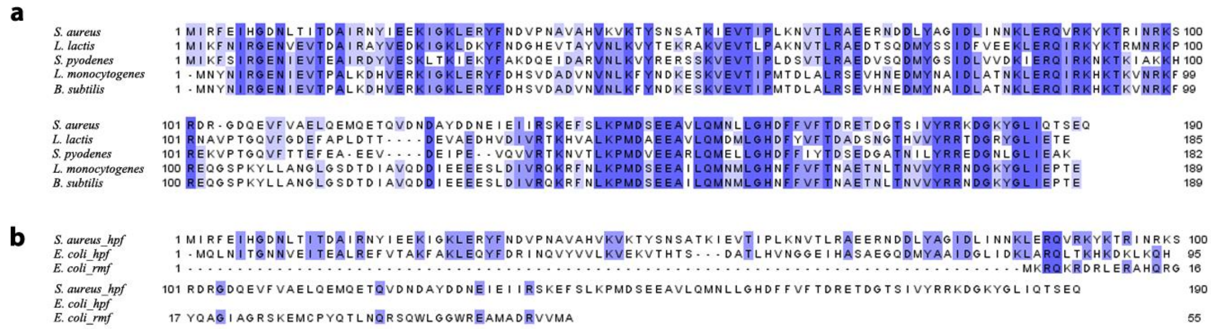

Supplementary Figure 1. Sequence alignment of HPF. A. Sequence alignment of HPF of *S. aureus* and other Gram-positive bacteria who's their 100S complexes have been studied shows high conservation (47%) among these bacteria. **B.** Sequence alignment of HPF_{SA}, HPF_{EC} and RMF_{EC} shows that HPF_{EC} and RMF_{EC} have some similarity to the N-terminal Part of HPF_{SA} and its center, respectively.

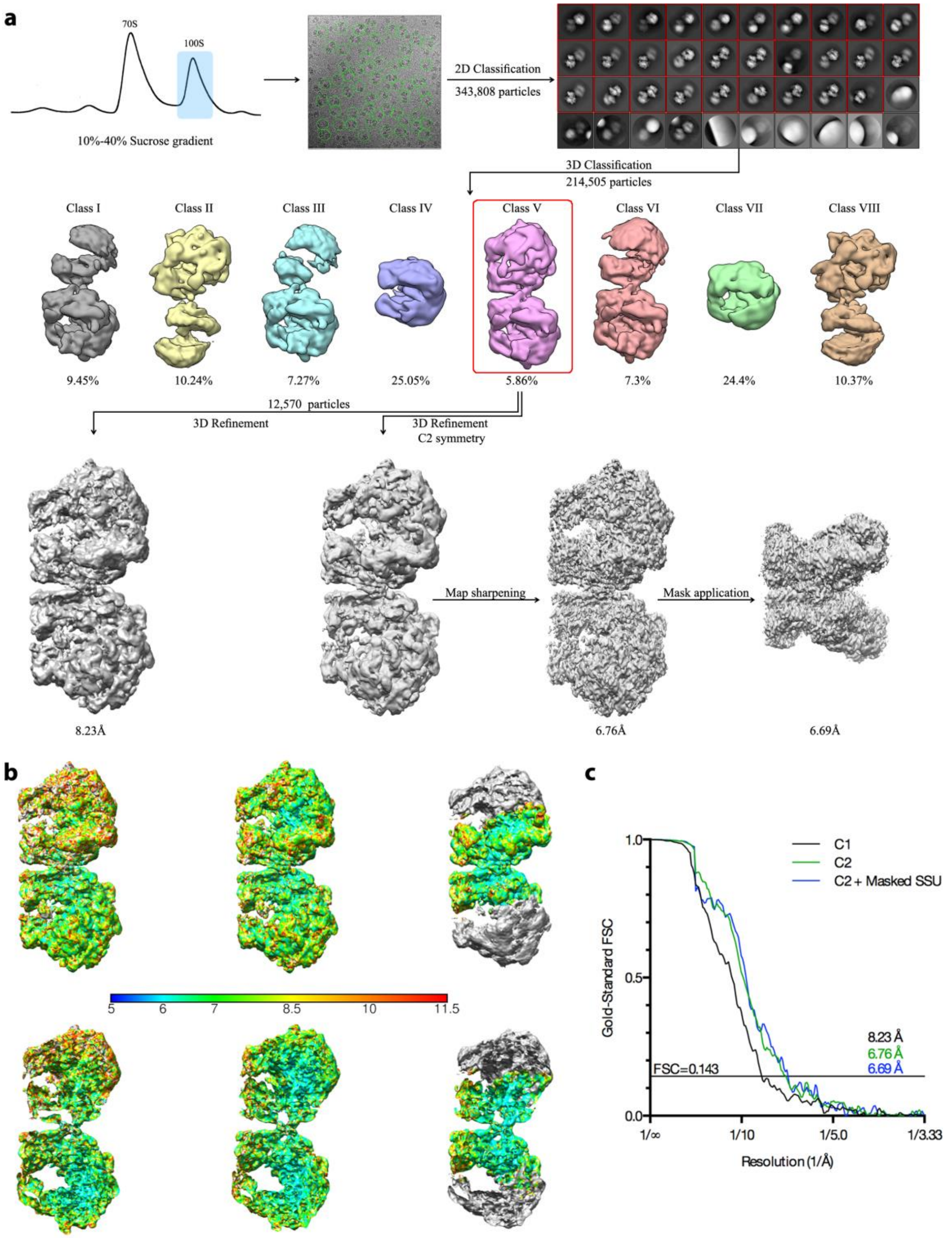

Supplementary Figure 2. Flow-chart for the cryo-EM analysis of SA100S. A. Cryo-EM image processing of the *S. aureus* 100S dimer. **B.** The local resolution map of the (from left to right) 100S, 100S processed with C2 symmetry, masked dimerization domain. **C.** Fourier shell correlation curves. Resolution of the calculated maps reported according to the gold standard FSC=0.143 criterion as implemented in RELION.

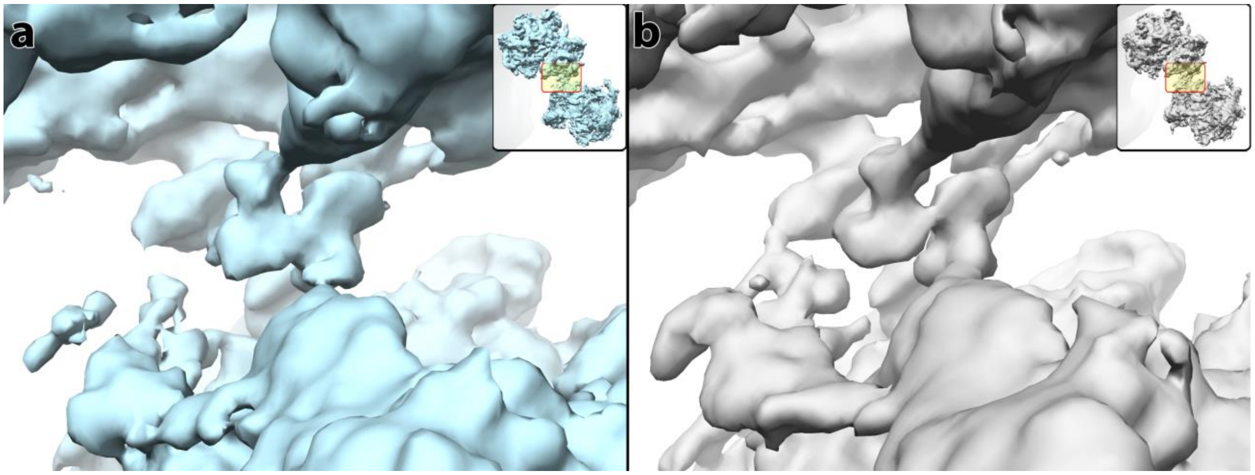

Supplementary Figure 3. C2 symmetry application improves the interface region within the SA100S maps. The interface region between the two 70S ribosome composing the 100S dimer in **A.** the SA100S dimer and **B.** the SA100S dimer refined with C2 symmetry applied. Both maps are displayed at the same σ. View of the whole 100S and the interface region within the 100S structure of both A and B. is shown in the upper right inset.

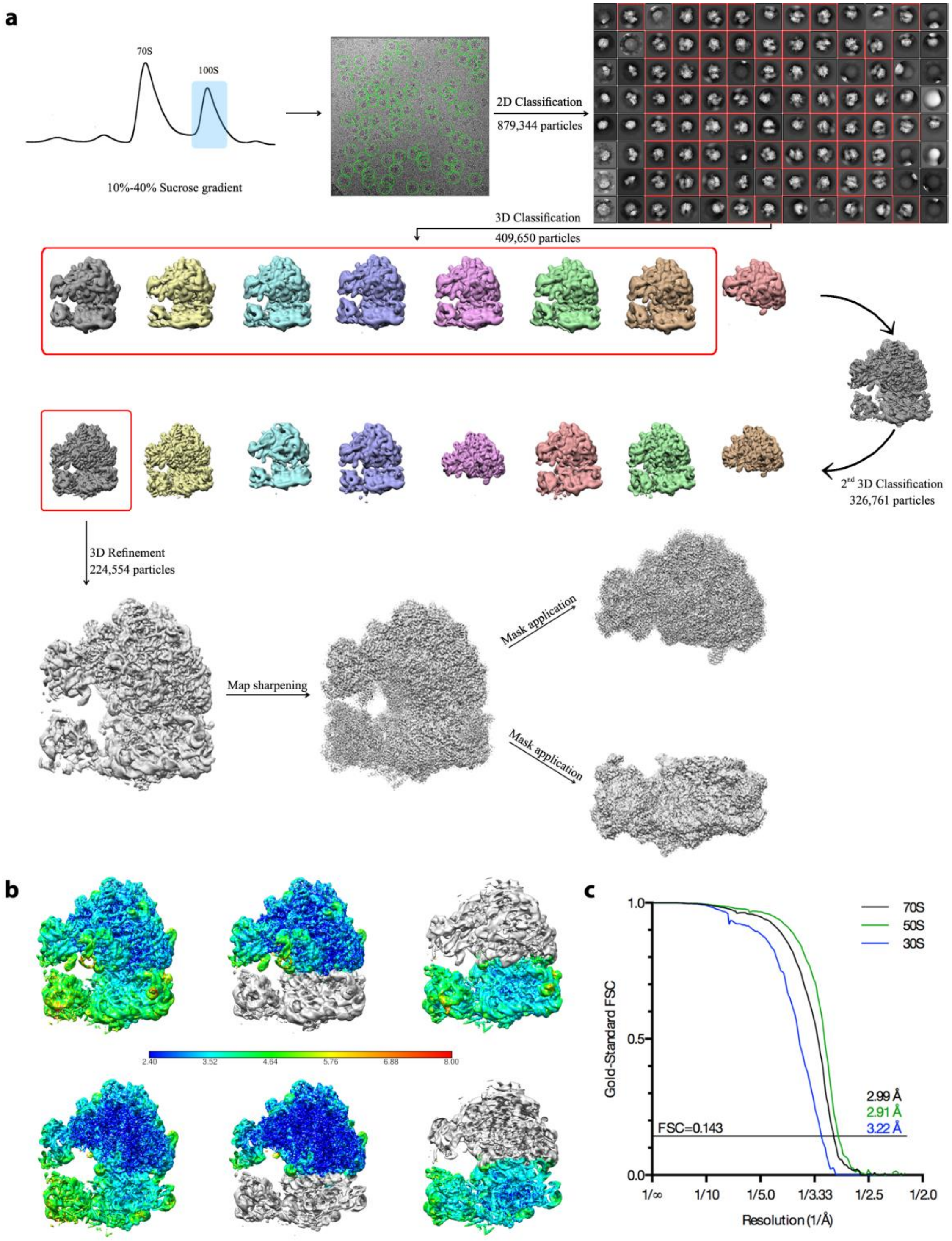

Supplementary Figure 4. Flow-chart for the cryo-EM analysis of SA70S composing the SA100S dimer. A. Cryo-EM image processing of the *S. aureus* 100S as monomers. **B.** The local resolution map of the (from left to right) 70S, 50S, 30S **C.** Fourier shell correlation curves. Resolution of the calculated maps reported according to the gold standard FSC=0.143 criterion as implemented in RELION.

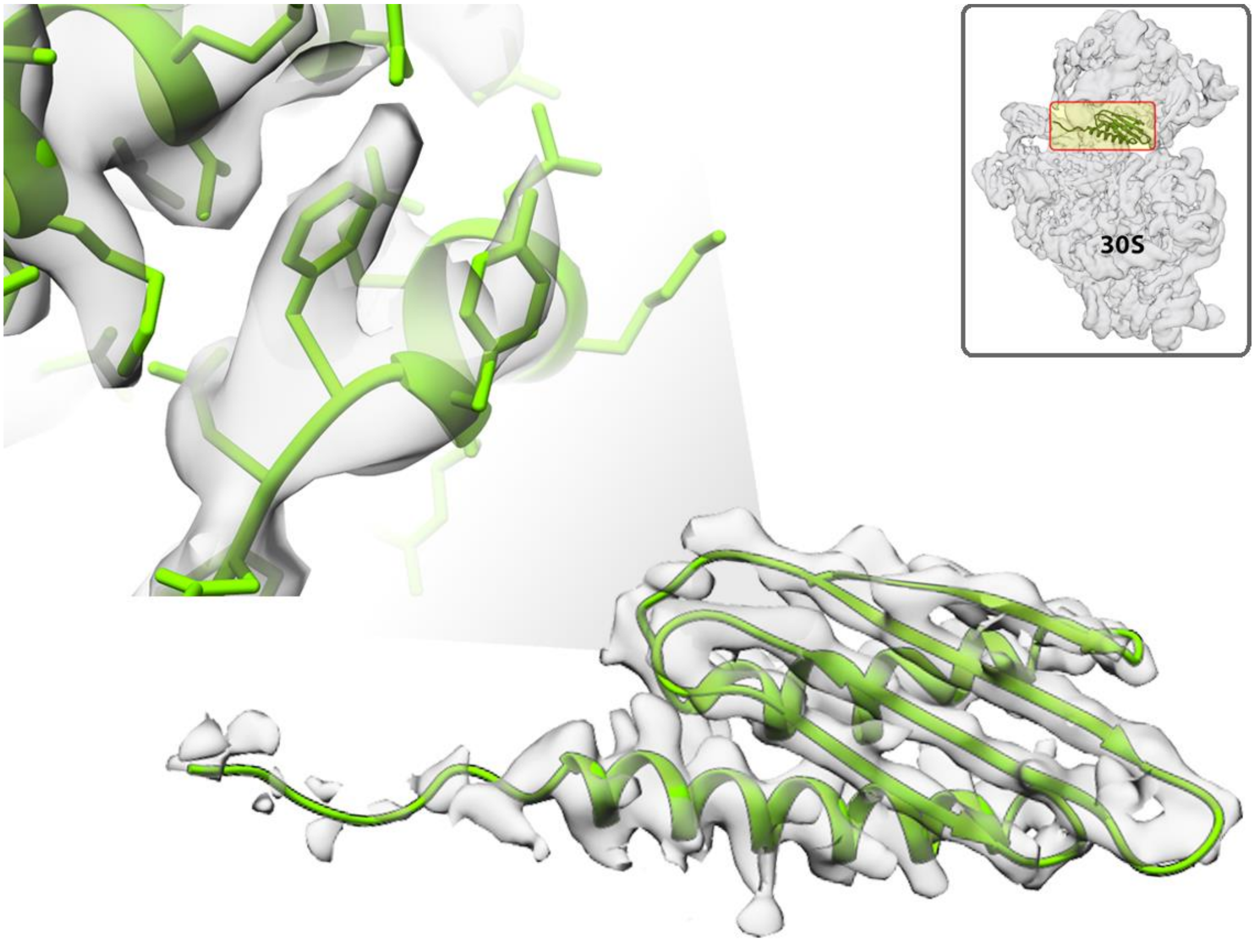

Supplementary Figure 5. N-terminal domain of HPF$_{SA}$. Bottom right, the whole N-HPF$_{SA}$, colored in chartreuse fitted into the 30S masked cryo-EM density map at 3.6Å. Top left, a close-up view into the protein. The N-HPF$_{SA}$ location on a single 70S monomer is shown in the upper right inset.

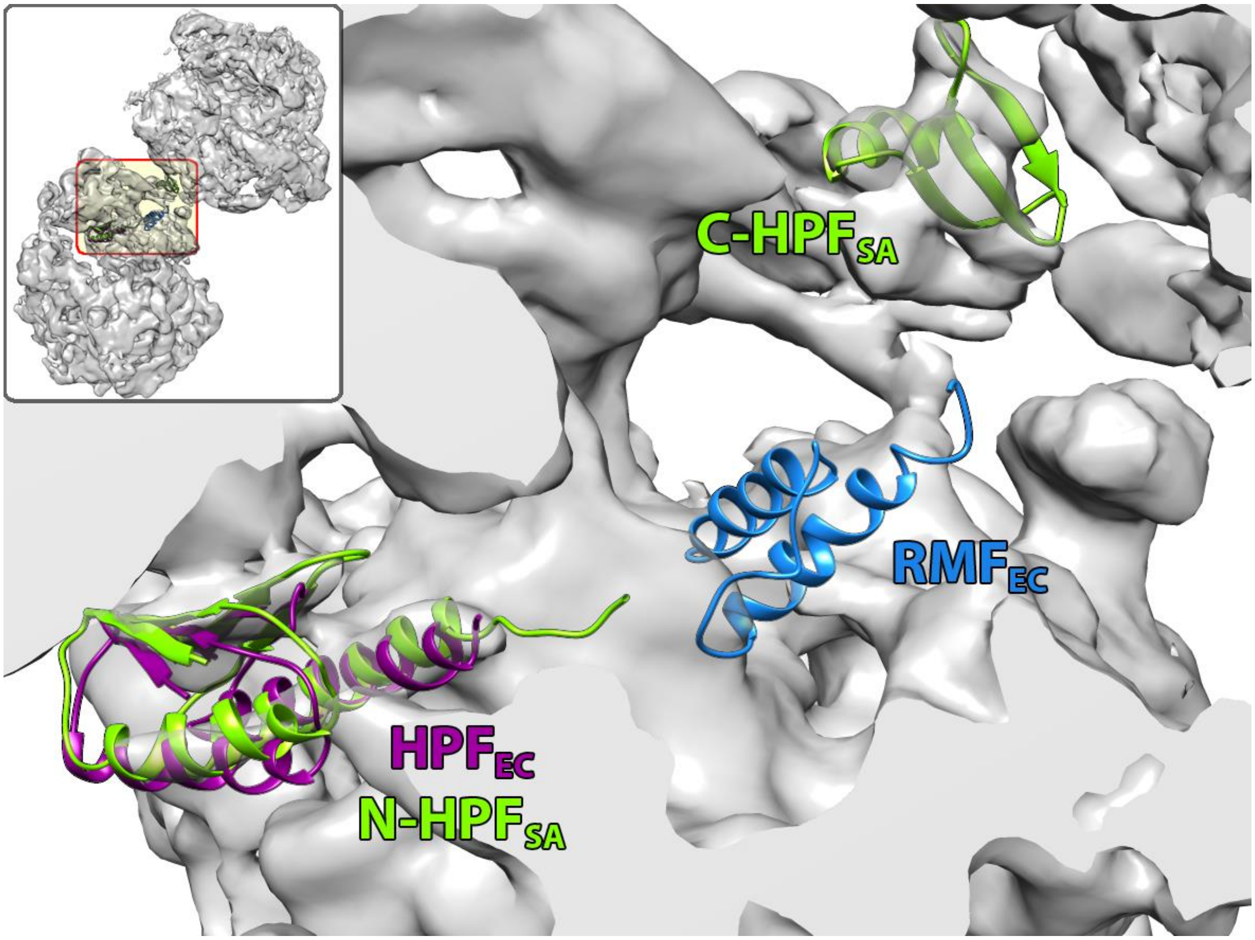

Supplementary Figure 6. Close-up view into HPF_{SA} binding pocket. HPF_{SA}, colored in chartreuse is fitted into the 6.7Å 100S cryo-EM map. Superposition of the structures of *Tth* bound to HPF_{EC} (magenta, PDB ID 4V8H) and RMF_{EC} (blue, PDB ID 4V8G) on the structure of SA100S reveals no additional density adjacent to the superimposed RMF_{EC}. The interface region within the 100S structure is shown in the upper left inset.

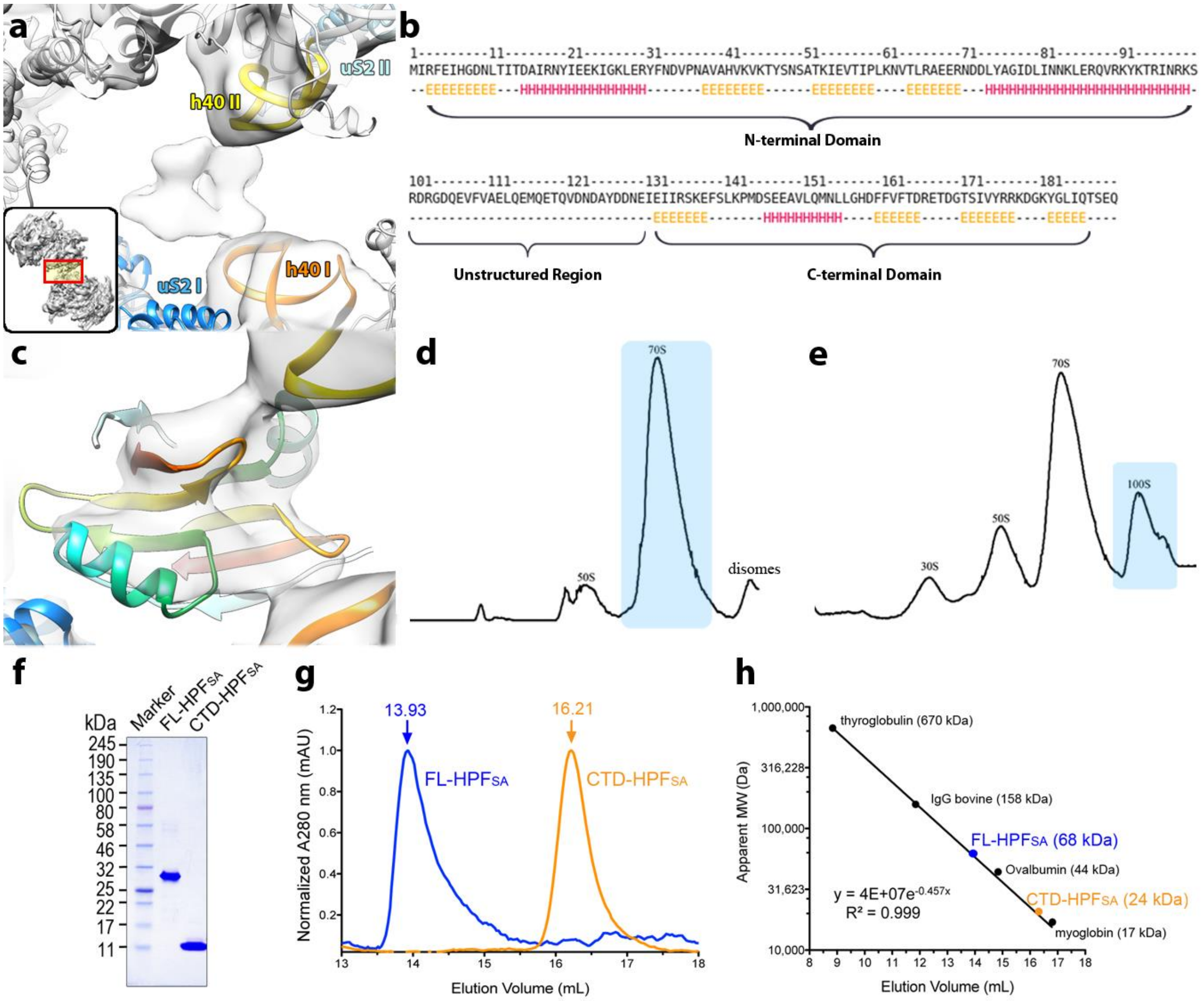

Supplementary Figure 7. C-terminal domain of HPF_{SA} analysis. A. Close-up view of the interface area of the two 30S subunits and the unidentified density of the 100S cryo-EM density map. The interface region within the 100S structure is shown in the lower inset. **B.** Secondary structure prediction of HPF_{SA} calculated by Jpred [1] suggests that this protein has two domains linked by a flexible loop. **C**. tertiary structure prediction of both C-HPF_{SA} calculated using SWISS-MODEL [2-4] and fitted into the unidentified density. **D, E.** Ribosome profile of C-HPF_{SA} truncated SA strain (**D**) and N-HPF_{SA} truncated SA strain (**E**). **F.** Coomassie blue-stained SDS-PAGE of the purified recombinant FL-HPF_{S}A and CTD-HPF_{SA}. The proteins were resolved on a 4-20% TGX (BioRad) gel. Each lane corresponds to ca. 10 μg of proteins. **G.** Elution profiles of the FL-HPF_{SA} (blue) and CTD-HPF_{SA} (orange). **H.** Standard curve for the molecular mass determination demonstrates the apparent molecular weight of FL-HPF_{SA} (68.8 kDa, eluted in 13.93 ml) and CTD-HPF_{SA} (24.2 kDa, eluted in 16.21 ml). This roughly corresponds to the dimer of the FL- HPF_{SA} (~30 kDa) and CTD- HPF_{SA} (~11 kDa) monomer, respectively, as seen in the SDS-PAGE gel.

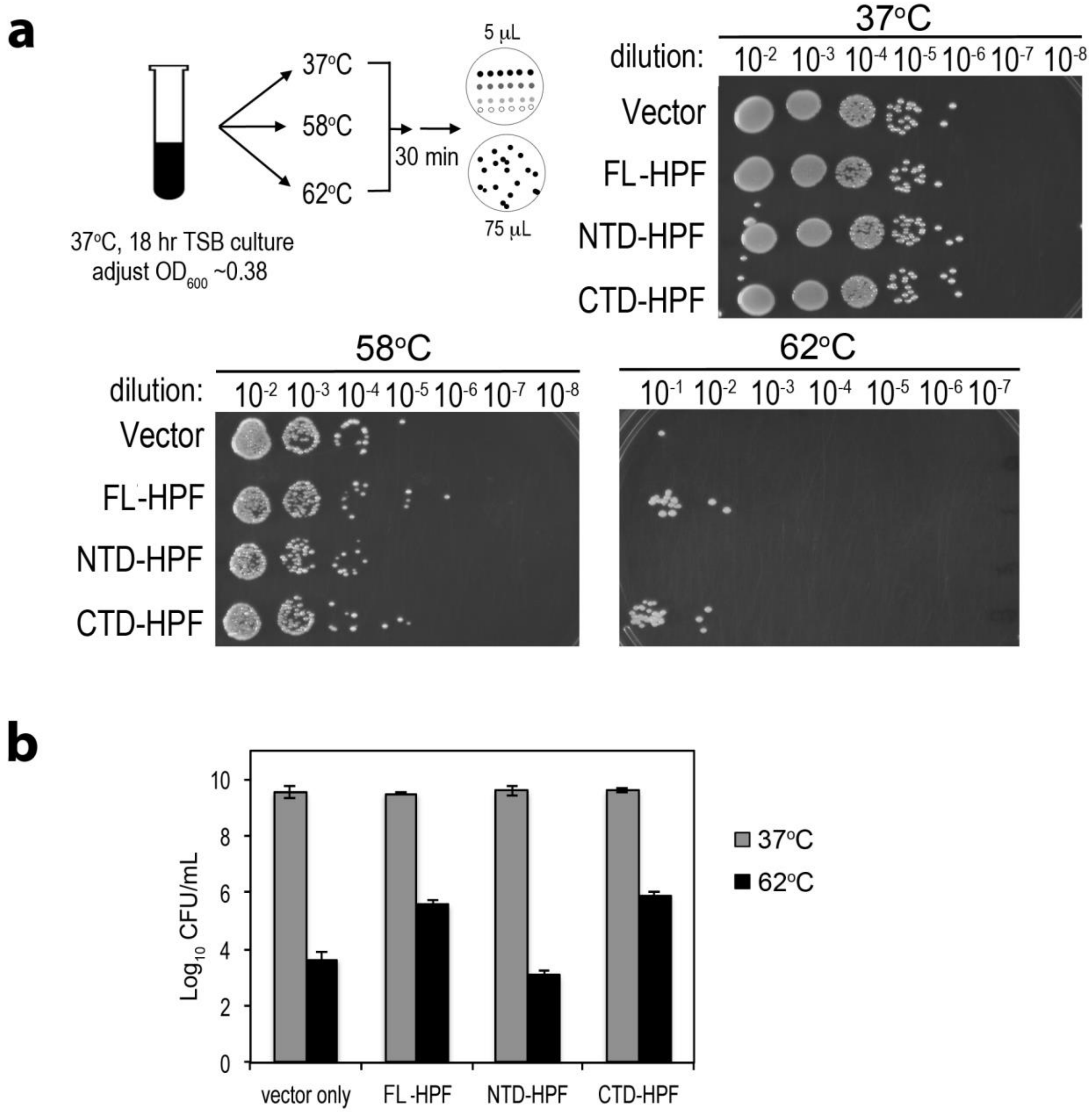

Supplementary Figure 8. Ribosomal dimerization is required for cell survival upon heat stress. A. Spotting assay. **B.** Determination of colony forming units (CFUs). S. aureus hpf knockout strains harboring the empty vector, full-length (FL) HPF, NTD-HPF and CTD-HPF were exposed to severe temperature upshift from 37°C to 58°C and 62°C for 30 min. Serially diluted cells were spotted (5 µL/spot) or spread (75 µL/plate) on agar plates and grown overnight at 37°C to determine cell viability. With the exception of 58°C (two replicates), spotting assay data are representative of three independent experiments. Thermal killing data in (B) are means and standard deviations (n=3). TSB, tryptic soy broth.

**Data collection**		
Microscope	Titan Krios	
Camera	CMOS (Falcon II)	
Voltage (kV)	300	
Magnification	75K	
Pixel size (Å.px^{-1})	1.07	
	**70S**	**100S**
Defocus range (µm)	0.4-3.6	0.4-4.4
Total dose (e/Å^2)	25	25
Dose per frame	~2.3	~2.3
Micrographs collected	5684	12,504
**Refinement**		
Number of particles (autopicked)	879,344	343,808
Number of particles (used for 3D reconstruction)	224,554	12,570
Resolution (Å; at FSC[a] = 0.143)	2.99	6.7
CC[a] (model to map fit)	0.69 [b]	0.88[c]
**RMS[a] deviation**		
Bonds (˚)	0.006	0.006
Angles (˚)	0.855	0.856
Chirality (˚)	0.046	0.046
Planarity (˚)	0.006	0.006
**Validation**[d]		
Clashscore[e]	7.6	7.81
Proteins		
MolProbity score	1.91	1.92
Rotamers outliers (%)	0.81	0.81
Ramachandran favored (%)	91.72	91.73
Ramachandran allowed (%)	8.26	8.25
Ramachandran outliers (%)	0.02	0.02
RNA		
Correct sugar pucker (%)	99.14	99.15
Correct backbone conformation	72.12	72.1

[a]FSC, Fourier shell correlation; CC, correlation coefficient; RMS, root-mean square.
[b]Only across atoms in the model; compiled using Phenix[5].
[c]Across whole map volume; compiled using Phenix[5].
[d]Compiled using MolProbity[6].
[e]Clashscore is the number of serious steric overlaps (>0.4 A˚) per 1000 atoms.

Supplementary Table 1. Data collection, refinement and validation statistics

Supplementary References

1. Drozdetskiy, A., et al., *JPred4: a protein secondary structure prediction server.* Nucleic Acids Research, 2015. **43**(W1): p. W389-W394.
2. Arnold, K., et al., *The SWISS-MODEL workspace: a web-based environment for protein structure homology modelling.* Bioinformatics, 2006. **22**(2): p. 195-201.
3. Biasini, M., et al., *SWISS-MODEL: modelling protein tertiary and quaternary structure using evolutionary information.* Nucleic Acids Research, 2014. **42**(W1): p. W252-W258.
4. Bordoli, L., et al., *Protein structure homology modeling using SWISS-MODEL workspace.* Nat. Protocols, 2008. **4**(1): p. 1-13.
5. Afonine, P., et al., *New tool: phenix. real_space_refine.* Computational Crystallography Newsletter, 2013. **4**: p. 43-44.
6. Chen, V.B., et al., *MolProbity: all-atom structure validation for macromolecular crystallography.* Acta Crystallographica Section D: Biological Crystallography, 2010. **66**(1): p. 12-21.
