# Supplementary material for: The cryo-EM structure of hibernating 100S ribosome dimer from pathogenic Staphylococcus aureus
Source: Nat Commun. 2017 Sep 28;8:723. doi: 10.1038/s41467-017-00753-8 (PMC5620080; doi:10.1038/s41467-017-00753-8)
Supplement: Supplementary file 3 — Description of Additional Supplementary Files [file 41467_2017_753_MOESM3_ESM.pdf]

Description of Additional Supplementary Files

File Name: Supplementary Movie 1

Description: **Structural differences of HPF_{SA} bound/unbound 30S subunits.** This movie demonstrates the conformational change of the head domain of the *S. aureus* 30S subunit induced by HPF_{SA} binding by morphing between the bound and unbound state. HPF_{SA} is colored in chartreuse and protein uS2 is colored in orange.
